# Understanding the Molecular Basis of 5-HT_4_ Receptor Partial Agonists through 3D-QSAR Studies

**DOI:** 10.3390/ijms22073602

**Published:** 2021-03-30

**Authors:** Alejandro Castro-Alvarez, Emigdio Chávez-Ángel, Ronald Nelson

**Affiliations:** 1 Laboratorio de Bioproductos Farmacéuticos y Cosméticos, Centro de Excelencia en Medicina Traslacional, Facultad de Medicina, Universidad de La Frontera, Av. Francisco Salazar 01145, Temuco 4780000, Chile; 2 Catalan Institute of Nanoscience and Nanotechnology (ICN2), CSIC and BIST, Campus UAB, Bellaterra, 08193 Barcelona, Spain; emigdio.chavez@icn2.cat; 3 Departamento de Química, Facultad de Ciencias, Universidad Católica del Norte, Av. Angamos 0610, Antofagasta 1270709, Chile

**Keywords:** Alzheimer’s disease, 5-HT_4_, partial agonist, 3D-QSAR, force and gaussian fields

## Abstract

Alzheimer’s disease (AD) is a neurodegenerative disorder whose prevalence has an incidence in senior citizens. Unfortunately, current pharmacotherapy only offers symptom relief for patients with side effects such as bradycardia, nausea, and vomiting. Therefore, there is a present need to provide other therapeutic alternatives for treatments for these disorders. The 5-HT_4_ receptor is an attractive therapeutic target since it has a potential role in central and peripheral nervous system disorders such as AD, irritable bowel syndrome, and gastroparesis. Quantitative structure-activity relationship analysis of a series of 62 active compounds in the 5-HT_4_ receptor was carried out in the present work. The structure-activity relationship was estimated using three-dimensional quantitative structure-activity relationship (3D-QSAR) techniques based on these structures’ field molecular (force and Gaussian field). The best force-field QSAR models achieve a value for the coefficient of determination of the training set of R^2^_training_ = 0.821, and for the test set R^2^_test_ = 0.667, while for Gaussian-field QSAR the training and the test were R^2^_training_ = 0.898 and R^2^_test_ = 0.695, respectively. The obtained results were validated using a coefficient of correlation of the leave-one-out cross-validation of Q^2^_LOO_ = 0.804 and Q^2^_LOO_ = 0.886 for force- and Gaussian-field QSAR, respectively. Based on these results, novel 5-HT_4_ partial agonists with potential biological activity (pEC_50_ 8.209–9.417 for force-field QSAR and 9.111–9.856 for Gaussian-field QSAR) were designed. In addition, for the new analogues, their absorption, distribution, metabolism, excretion, and toxicity properties were also analyzed. The results show that these new derivatives also have reasonable pharmacokinetics and drug-like properties. Our findings suggest novel routes for the design and development of new 5-HT_4_ partial agonists.

## 1. Introduction

Alzheimer’s disease (AD) is a neurodegenerative disorder that mainly affects people over 60 years old. The current pharmacotherapy only provides palliative treatments, reducing the associated symptoms through the increase of cholinergic function. This pharmacotherapy can produce unwanted side effects such as abdominal pain, muscle cramps, tremors, and fatigue, among others [1]. In this sense, there is a need for new therapeutic targets for the treatment of this disorder.

The 5-HT_4_ receptor (5-HT_4_R) belongs to a superfamily of G-protein coupled receptors (GPCRs) [2,3,4]. This receptor is highly expressed in the brain regions of the hippocampus, amygdala, and cerebral cortex, areas of the brain related to short- and long-term memory and cognitive processing, so that deterioration of this region would be associated with neurological diseases such as Alzheimer’s disease [5,6]. The 5-HT_4_R has been reported to play an essential role in disorders of the central nervous system (CNS) such as AD [7,8], peripheral nervous system (PNS) disorders [9], irritable bowel syndrome [10,11,12], and gastroparesis [13,14,15]. Moreover, 5-HT_4_R agonists modulate peptides derived from the soluble amyloid precursor protein-α (a non-amyloidogenic protein) that plays a role in neuroprotection against the neurotoxic effects of β-amyloid [16]. Therefore, 5-HT_4_R partial agonists show very promising activity for symptomatic treatments of cognitive disorders in AD [17]. Its dual mechanism of action in treating AD and other cognition-related diseases makes 5-HT_4_R a very attractive target for new drug discovery. Consequently, several structurally diverse heteroaromatic compounds [18,19,20,21] have been explored as 5-HT_4_R total or partial agonists for both CNS and PNS. Nirogi et al. reported a series of 5-HT_4_R compounds with 3-isopropylimidazo [1,5-a]-pyridine-carboxamide scaffold, most of which showed cognition-enhancing properties in animal models [22]. However, their absorption, distribution, metabolism, excretion, and toxicity (ADMET) properties were not satisfactory due to their low ability to penetrate the blood-brain barrier. Their results revealed that these molecules are composed of an aromatic fragment, a coplanar functional group, and a bulky substituent. Recently, Nirogi reported new 5-HT_4_R partial agonists with good ADMET properties and potential drug candidates [23].

To design new 5-HT_4_R agonists, theoretical studies are substantially essential to expedite and save resources. Several computational methods simplify the drug discovery process. Quantitative structure-activity relationship (QSAR) is a ligand-based drug design method, which relates to the biological activity of compounds with several physicochemical properties [24]. However, QSAR techniques have limited efficacy for designing new functional molecules due to the lack of three-dimensional (3D) molecules’ structures. Consequently, 3D-QSAR averts this problem by using the 3D-attributes of ligands and chemometric tools. That significantly improves the predictability of the biological activity of the model [25,26,27,28].

In this work, we present a computational study of a three-dimensional quantitative structure-activity relationship (3D-QSAR) of a set of molecules with agonist activity on 5-HT_4_ receptors. The calculations were carried out by using force- and Gaussian-field based QSAR models. Our 3D-QSAR study aims to obtain helpful information to guide future 5-HT_4_R agonists’ design with promising therapeutic activity and that these new analogues have good ADMET properties as prospective drug candidates.

## 2. Results and Discussion

### 2.1. Studied Compounds

**Figure 1:**
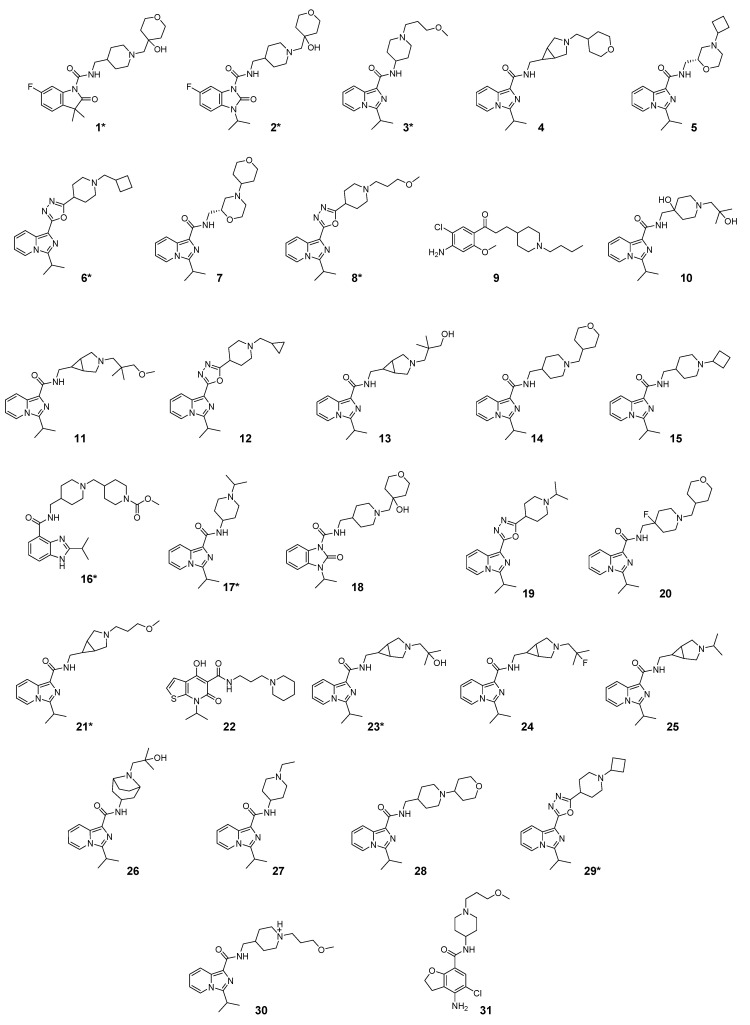

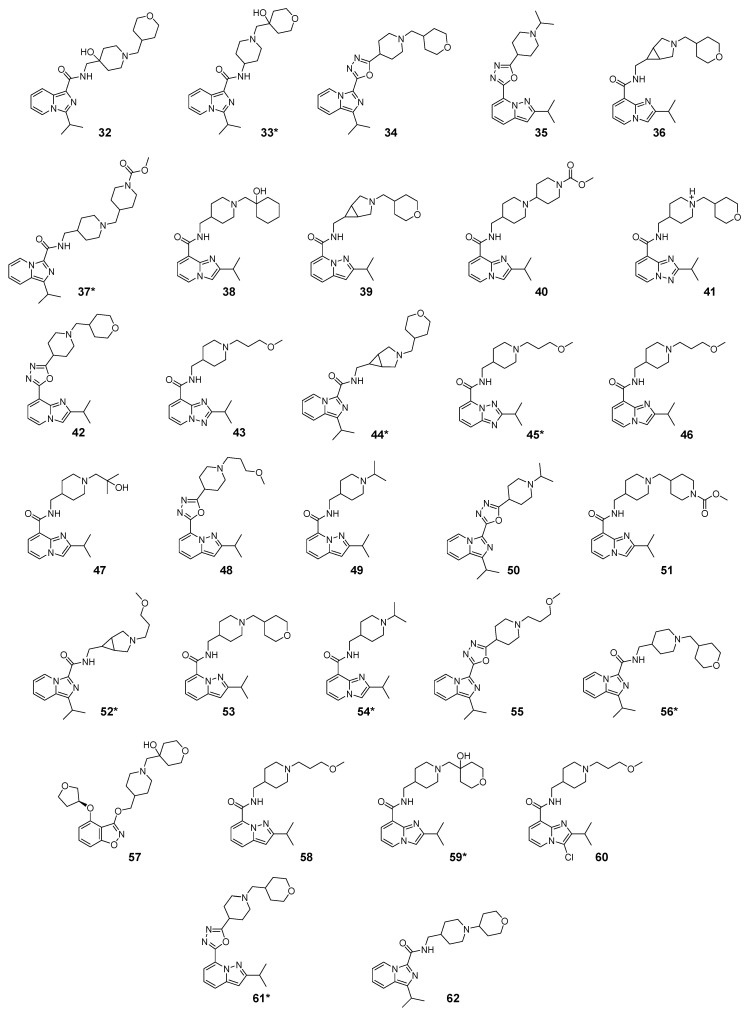
Dataset of 62 compounds. Structures with an asterisk (*) were used as a test dataset.

The studied dataset was based on Brodney et al. [18] and Nirogi et al. [22,23]. They reported different compounds with biological activity (5-HT4 receptor partial agonist.) expressed in EC_50_ in nanomolar concentration (see Table S1 of the Supplementary Material). In total, 62 compounds were divided in training (43 compounds) and test dataset (19 compounds), as is shown in Figure 1. The biological activity was expressed in terms of pEC_50_ for this study.

Finally, the ADMET properties were estimated by using the pkCSM [29,30] and SwissADME [31,32] web services. On one hand, pkSCM calculates the pharmacokinetic properties using structural similarity of the new molecules with molecules with known pharmacokinetic properties and, from this comparison, the pkSCM program returns estimated values for the new molecules. On the other hand, SwissADME calculates the different drug-likeness parameters by calculating physicochemical descriptors for each of the designed molecules. These descriptors are lipophilic, water-solubility, surface volume, among others (an extended description can be found in Section S3 of the Supplementary Material). From these descriptors, the program generates an estimate of drug-likeness based on parameters from Lipinski [33], Ghose [34], Veber [35], and Egan [36].

### 2.2. Statistical Results

**Figure 2:**
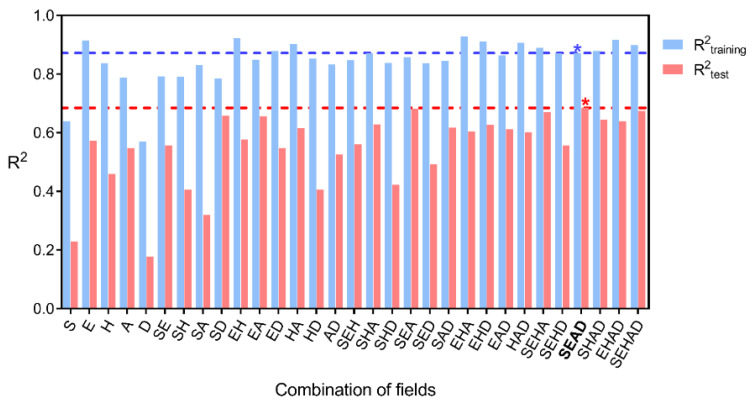
The results of the distribution of R^2^_test_ values were obtained from 31 combinations of Gaussian fields. S, steric; E, electrostatic; H, hydrophobic; A, hydrogen-bond acceptor; D: hydrogen-bond donor. The best Gaussian fields combination is highlighted with an asterisk on the bars with R^2^_training_ 0.898 and R^2^_test_ 0.695.

The statistical results for force- and Gaussian-field QSAR (FFQSAR and GFQSAR, respectively) are presented in Table 1 and Table 2. All possible field combinations were tested for both FFQSAR and GFQSAR. In the case of FFQSAR, the combination of the steric and electrostatic fields was statistically significant (see Table 1) with R^2^_training_ of 0.821 and R^2^_test_ 0.667. The GFQSAR models with the highest R^2^_test_ values were those that considered the field combinations (see Figure 2 and Table 2). The best model with the highest R^2^_training_ and R^2^_test_ was chosen for the study. This model presented steric (0.420), electronic (0.125), acceptor (0.304), and donor hydrogen-bond (0.151) contribution, with a correlation between experimental and predicted data showing R^2^_training_ 0.898 and with an external validation 0.695 (R^2^_test_) (see Table S2). The experimental activities, the predicted values, and the residual values for this model are shown in Table 3. All the compounds showed low residual values with a range from −1.1 to 1.5 for FFQSAR and −1.2 to 1.2 for GFQSAR.

A detailed discussion about the best FFQSAR and GFQSAR models’ internal and external validation parameters is presented in Section S2.4 of Supplementary Material.

### 2.3. Analysis of the 3D-QSAR Models

The dataset of 62 compounds was randomly separated into a training set (43 composites) and a test set (19 composites). The training set was used to run different 3D-QSAR models with FFQSAR and GFQSAR (different field combinations). The best models were evaluated with the test set. The visualization of the best 3D-QSAR models were analyzed by recognizing the colored regions highlighting the favored and disfavored areas that explain the 5-HT_4_R partial agonist activity of the different compounds studied. For FFQSAR, the best model was achieved with the combination of steric and electrostatic fields with R^2^ 0.821 for the training set and R^2^ 0.667 for the test set, respectively.

#### 2.3.1. Force-Field Based 3D-QSAR Model—Steric and Electrostatic Contour Map

**Figure 3:**
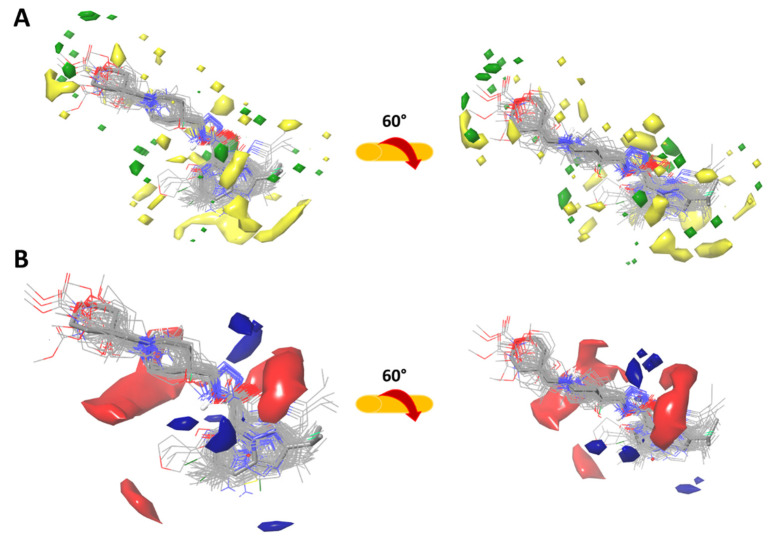
(**A**) Steric contour and (**B**) electrostatic contour maps for the best force-field based 3D-QSAR model. The active molecules are shown in sticks for (**A**) and (**B**), respectively. The favorable and disfavored regions of the steric field shown in (**A**) are highlighted in green and yellow, respectively, while the electrostatic regions are shown in (**B**), the favorable electropositive and unfavorable electronegative regions are highlighted in blue and red, respectively.

Green and yellow colors represent the force field-based steric interactions. The green and the yellow regions represent zones where bulky substituents’ addition can increase or decrease activity, respectively (see Figure 3A). In the most active molecule (compound **53**) the green contours surround the tetrahydropyran and the heterocyclic aromatic ring. In contrast, the less active compounds have an aromatic ring, whose orientation is in the yellow regions, resulting in decreased biological activity.

Figure 3B shows field-based electrostatic interactions which are represented by red (electronegative) and blue (electropositive) contours. The regions coloured in blue and red represent the most influential electropositive and electronegative zones in biological activity. One of the largest regions for electronegative interactions is around the tertiary amine of the piperidine and the amide’s carbonyl. Moreover, electropositive regions corresponding to the nitrogen atoms of the oxadiazole ring are present in some compounds. The less active molecules of the dataset have a disfavored conformation due to the imidazo[1,5-*a*]pyridine heterocyclic ring. We observed that all compounds with low partial 5-HT_4_R agonist activity had a chain length from amide to morpholine N of three carbon atoms. It suggests that this distance is responsible for the decrease in biological activity.

#### 2.3.2. Gaussian-Field Based 3D-QSAR Model—Steric Contour Map

The GFQSAR was generated using five-factors partial least squares (PLS) and correlating four fields: steric (S), electrostatic (E), hydrogen bond donors (HBD), and hydrogen bond acceptor (HBA). A Q^2^ value of 0.886 was derived from the leave-one-out (LOO) cross-validation method. A non-cross-validation analysis yielded an R^2^ = 0.898 with a standard error of estimate SD = 0.377 and F ratio of 360.13 (see Table S2). The steric, electrostatic, HBA, and HBD fields contributions (ranging from 0 to 1) were 0.420, 0.125, 0.304, and 0.151, respectively (see Table 2). The field contributions of steric (0.420) and HBA (0.304) intensities were higher than the electrostatic (0.125) and HBD (0.151), indicating a larger requirement of steric and hydrogen-bond acceptor for protein-ligand interactions.

**Figure 4:**
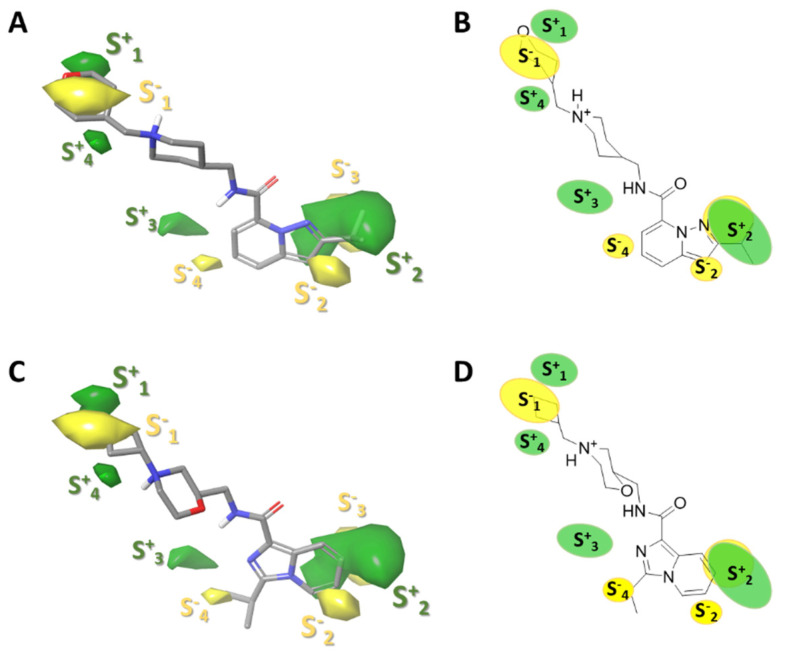
Contour maps obtained for the best Gaussian-based 3D-QSAR model steric (hydrogen bond donors, HD), green and yellow regions indicate a favorable and unfavorable steric interaction, respectively. An active molecule (**53**) is represented in (**A**) (sticks representation) and (**B**) (as draw representation). Less active molecule (**5**) is shown in (**C**) (sticks representation) and (**D**) (as draw representation).

The steric interactions represented in green and yellow contour are shown in Figure 4. The green regions of the molecules represent the favorable effect of the bulky substituents, i.e., at these positions, the bulky groups will have higher activity. Conversely, the yellow outlines represent the regions where the bulky groups will reduce activity.

For case **53** (Figure 4A,B), the S^+^_2_ contour (green contour) shows a favorable activity closer to the isopropyl of the aromatic ring. This trend was also observed for the highly active molecules **38**, **58**, and **59**. On the other hand, an opposite behavior was observed for moderately and less active molecules with substitutions with different orientations on the aromatic ring, as is compound **5** (Figure 4C,D), a molecule that shows a deficient biological activity, whose isopropyl orientation is opposite to that of compound **53**. Similar behavior was also observed for compounds **45**, **43**, and **20** with imidazo[1,5-*a*]pyridine ring.

#### 2.3.3. Gaussian-Field Based 3D-QSAR Model—Electrostatic Contour Map

**Figure 5:**
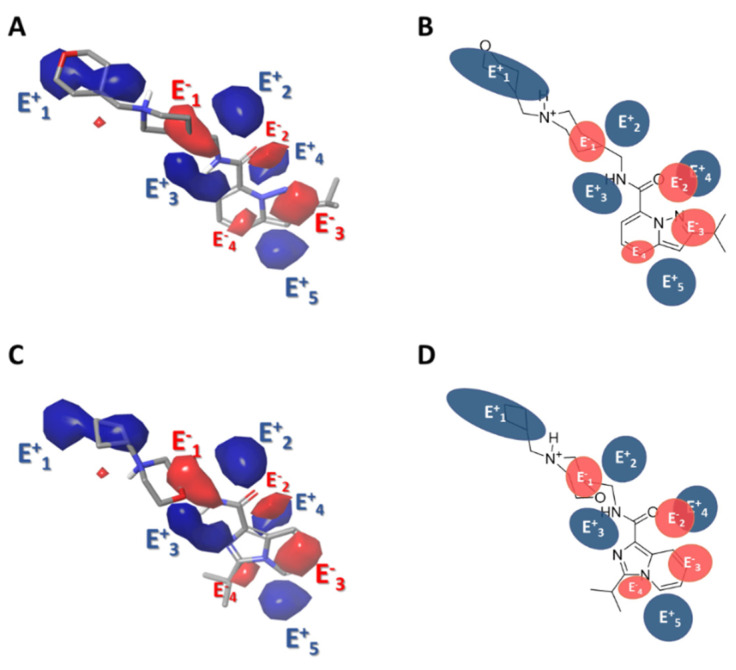
Contour maps obtained for the best Gaussian-based 3D-QSAR model with electrostatic interaction (HD), blue and red regions indicate favorable electropositive and electronegative interactions, respectively. Active molecule (**53**) is represented in (**A**) (sticks representation) and (**B**) (as draw representation). Less active molecule (**5**) is represented in (**C**) (sticks representation) and (**D**) (as draw representation).

As for the electrostatic contour maps, the blue (E^+^_1_, E^+^_2_, E^+^_3_ and E^+^_4_) and red (E^−^_1_, E^−^_2_, E^−^_3_ and E^−^_4_) contours represent the favorable and unfavorable components of the electrostatic field (Figure 5). The bulky E^+^_1_ contour surrounding the tetrahydropyran ring (Figure 5A,B) provides information on analogues that have electron-donating substituents that favor biological activity, such as compounds **18**, **38,** and **59** (pEC_50_ 9.301, 9.523, and 9.398, respectively), compounds that have a tertiary hydroxyl group on the tetrahydropyran ring (compounds **18** and **58**). The electropositive E^+^_3_ contour highlights the importance of amide hydrogen for biological activity (contour that reappears in the study of hydrogen-bonding donor groups, Section 2.3.5).

The electronegative E^−^_3_ and E^−^_4_ contours highlight the importance of the nitrogen specificity of the aromatic ring that would provide electron density and answer biological activity; however, the E^−^_3_ contour stands out (Figure 5A,B), in proportion, more than the E^−^_4_ contour, therefore, compounds that have a nitrogen in E^−^_3_ are more active than those that have a nitrogen in E^−^_4_ (Figure 5C,D). Finally, the electropositive contours E^+^_2_ and E^+^_4_ would respond to compounds with 1,3,4-oxadiazole groups; however, their biological activity is moderate to low (**61** with pEC_50_ 7.678, **48** with pEC_50_ 7.208 and **35** with pEC_50_ 7.268, see Table 3).

#### 2.3.4. Gaussian-Field Based 3D-QSAR Model—Hydrogen Bond Acceptor Contour Maps

**Figure 6:**
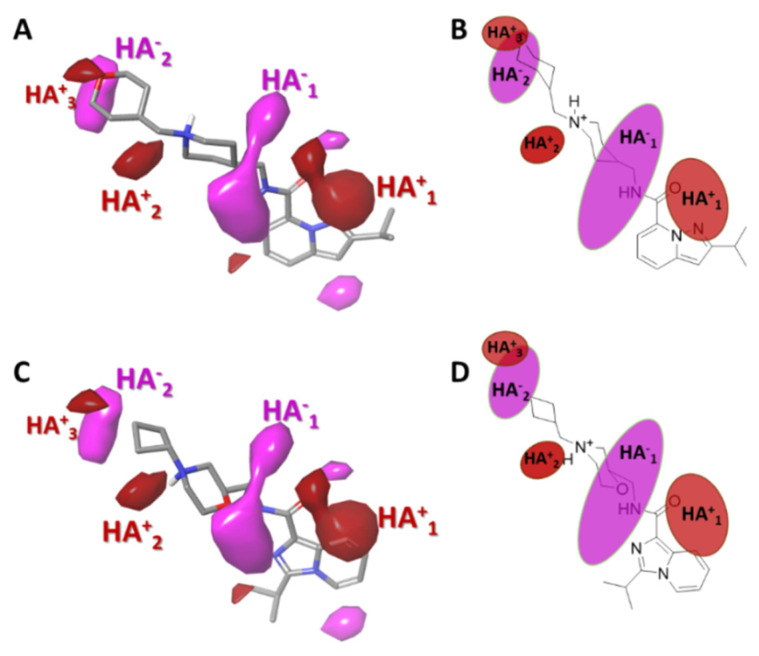
Contour maps obtained for the best Gaussian-based 3D-QSAR model hydrogen bond acceptors (HBA), red and magenta regions indicate a favourable and unfavourable hydrogen bond donor interaction, respectively. The active molecule (**53**) is represented in (**A**) (sticks representation) and (**B**) (as draw representation). The less active molecule (**5**) is represented in (**C**) (sticks representation) and (**D**) (as draw representation).

Hydrogen bond acceptor functional groups provide properties that determine the biological activity of a drug candidate compound. Therefore, the contour map obtained with the GFQSAR model establishes which HBA regions would help in the biological activity of the molecule. Figure 6 shows the impact of the HBA groups on the 5-HT_4_R partial agonist activity of the molecules. The regions of highest and lowest affinity are shown with red and magenta contours, respectively. In general, in all the studied molecules, the tetrahydropyran group is responsible for establishing hydrogen bonds (HA^+^_3_ contour). The large affinity region (HA^+^_1_ contour) near the amide carbonyl suggests that it favours 5-HT_4_R partial agonist activity. In contrast, fewer affinity regions indicate that functional groups attached directly to the amide, such as hydroxyl on the piperidine ring or replacing the piperidine with morpholine will reduce biological activity such as compounds **5**, **10**, and **32** with EC_50_ of 5.648, 7.141, and 7.246 (see Table 3), respectively. That finding is represented by a large magenta colored area (HA^−^_1_).

#### 2.3.5. Gaussian-Field Based 3D-QSAR Model—Hydrogen Bond Donor Contour Maps

**Figure 7:**
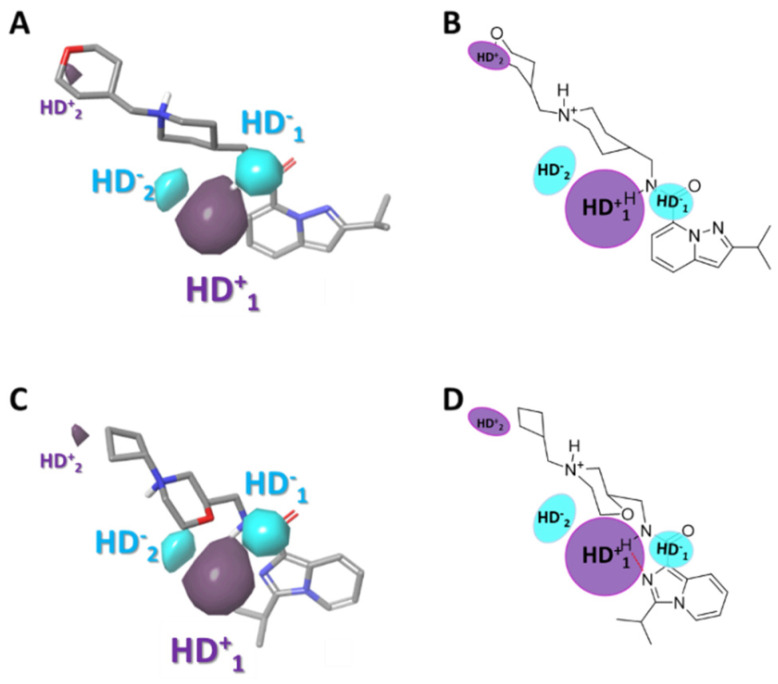
Contour maps obtained for the best Gaussian-based 3D-QSAR model hydrogen bond donors (HD), violet regions indicate favorable hydrogen bond donor interactions and cyan regions indicate unfavorable hydrogen bond donor interactions. The active molecule (**53**) is represented in (**A**) (sticks representation) and (**B**) (as draw representation). The less active molecule (**5**) is represented in (**C**) (sticks representation) and (**D**) (as draw representation).

The contour of hydrogen bond donor (HBD) maps provides significant information about the functional groups involved in the biological activity in these compounds. Figure 7 shows the favorable (purple) and unfavorable (cyan) HBD regions. The favorable contour (HD^+^_1_) highlights the hydrogen atom of the amide, suggesting that HBDs are favored at that position and explaining why molecules with 1,3,4-oxadiazole have less 5-HT_4_R partial agonist activity (compounds **8**, **12**, **19**, **34**, **35**, **42**, **48**, **50**, **55**, and **61**; EC_50_ values between 6.067 to 7.678), due to the change of the hydrogen bond donor capacity of the amide by the hydrogen bond acceptor group such as the oxadiazole ring.

### 2.4. Design of New Derivatives

**Figure 8:**
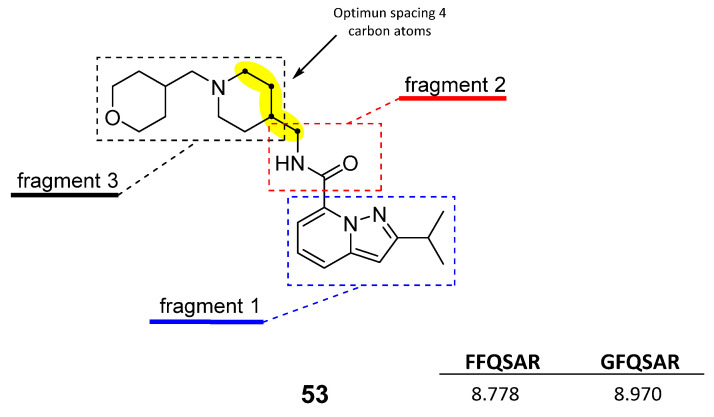
Template molecule (**53**) used for the derivation of new molecules/compounds with enhanced bioactivity.

Based on the results of the 3D-QSAR studies, thirty-nine new compounds (see Table S3, Supplementary Material) were designed and evaluated using molecule **53** as a template. Ten new 5-HT_4_R partial agonists with bioactivity greater than that predicted from the template molecule were selected using the GFQSAR model from these thirty-nine compounds. The structures of the ten new designed compounds and their pEC_50_ values predicted by the constructed FFQSAR and GFQSAR models are shown in Table 4. The design of these new derivatives seeks to enhance the steric (S^+^_1_), electrostatic (E^+^_1_), and hydrogen bond acceptor (HA^+^_3_ and HA^+^_2_) regions by modifying fragment 3 of the template molecule (see Figure 8). All the proposed molecules have a predicted activity better than the template molecule (**53**, pEC_50_ = 8.970) according to the GFQSAR model. However, only compound **var8** showed higher activity than **53** using both models.

In general, the presence of the pyrazolo[1,5-*a*]pyridine ring provides more active analogues. This aromatic heterocyclic system provides lipophilic and electronic features. Furthermore, the amide attached to the heterocyclic ring at position 7 has higher biological activity. On the other hand, all the analogues have a fragment 3 whose extension is four carbon atoms from the amide’s nitrogen to the heterocycle’s nitrogen except compound **var8**, which has a secondary aliphatic amine. This extension of fragment 3 provides good biological activity, where **var1**, **var2**, **var3**, **var6**, **var7**, and **var9** have an aliphatic ring that favors the steric region (green color, see Figure 3) of the 3D-QSAR models. These rings have at least one functional group with hydrogen bond acceptor properties favoring the HA^+^_3_ regions’ interaction (see Figure 6). In contrast, **var4**, **var5**, **var8**, and **var10** compounds have an aliphatic chain with an oxygenated functional group interacting in the HA^+^_2_ hydrogen bond acceptor region.

### 2.5. ADMET Predictions

Most drug candidates fail to make it through clinical trials in the drug discovery process because of their poor pharmacokinetics. To assess whether all newly designed compounds could become potential drugs, we perform ADMET predictions.

ADMET properties are shown in Table 5, and drug similarity predictions are shown in Table 6. The intestine is the main site of absorption of an orally administered drug. A molecule with an absorbance of more than 30% is considered well absorbed. As shown in Table 5, the intestinal absorbance of ten molecules is between 92.7% and 96.3%, which reveals a very good absorption in the human gut. Significantly, the intestinal absorbance of compound **var6** is higher than 96%. A volume of distribution (VDss) greater than 0.45 is considered high. High VDss indicates that more drugs are distributed in tissues than in plasma. The VDss of the ten new compounds were higher than 0.45.

Metabolism plays an essential role in converting pharmacological compounds. Cytochromes CYP2D6 and CYP3A4 are the two main P450 isoforms responsible for drug metabolism. As indicated in Table 5, all designed small molecules were neither substrate nor inhibitor of CYP2D6, a feature that may be an advantage since compound **53** is inhibitory to CYP2D6. Furthermore, the compounds were substrates of CYP3A4 except for compounds **var8**, **var9**, and **var10**, indicating that CYP3A4 can metabolize compounds **var1**-**var7**. Compounds **var2**, **var3**, **var4**, **var5**, **var6**, **var7**, and **var8** are not CYP3A4 inhibitors, implying that they will not affect normal drug metabolism.

Drug clearance related to bioavailability is essential in determining dosing rates to achieve steady-state concentrations in the body. From the predicted total clearance, all compounds can be excreted without problems at the renal level.

Furthermore, drug toxicity is another important index for drug screening. Drugs should be as non-toxic to human health as possible or have a wide therapeutic margin. All new derivatives are non-toxic to AMES (estimation of the mutagenic potential of chemical compounds) and do not cause skin sensitization. However, all new compounds have potential hepatotoxicity, which could possibly alter normal liver function. To further understand this unfavorable side effect, the synthesis of the proposed compounds must be tested in a living organism. However, such studies go beyond the current stage of this study.

Finally, the online tool SwissADME (http://www.swissadme.ch/, accessed on 19 February 2021), which provides access to several different rule-based filters, was used to predict drug similarity. As shown in Table 6, all new compounds meet the Lipinski and Egan drug similarity rules; only compounds **var1** and **var9** fail the Ghose filter, and compound **var5** fails the Veber filter. According to Lipinski and Egan’s drug similarity rules, the results of multiple evaluations indicate that these computationally designed compounds can be converted into oral drugs. The synthetic accessibility values of all designed molecules are approximately 4, meaning that they are synthesizable compounds (synthetic accessibility ranges from 1–10).

## 3. Materials and Methods

### 3.1. Dataset Collection

A total of 62 partial agonists of the 5-HT_4_ receptor (Figure 1), which showed promissory potency, were collected from the literature [18,22,23]. All the compounds with pEC_50_ values ranging from 5.64 to 10.0 were used in this study. The geometry for all these molecules was converted into a 3D structure using OCHEM. The 3D structure of the molecules was processed with OMEGA [37] module using the following parameters: (i) AM1_BCC Force field, (ii) FixpKa from the QUAPAC package for all possible ionization states at a given biological pH, (iii) one low energy conformation per ligand. Force- and Gaussian-field 3D-QSAR calculations were performed for all the molecules. All the training and test set molecules with experimental and predicted EC_50_ values were listed in Table S1 of the Supplementary Material.

### 3.2. Alignment

The alignment of molecules is the most crucial input for the generation of 3D-QSAR models. The compound with the highest activity (**53**) was used as the template molecule. A shape-based alignment was used for all conformers of each ligand. These alignments were carried out with ROCS suite [38]. Finally, each ligand’s best conformer was filtered considering electrostatic field compound **53,** as is shown in Figure S1 of the Supplementary Material.

### 3.3. Field-Based QSAR Model

The 3D-QSAR analysis using field-based methods was performed with the QSAR tool of the Schrodinger Suite. The 3D-QSAR method builds the model by relating the known activities and molecular elements of the training set using the OPLS_2005 force field. The steric and electrostatic field around the ligand on a 3D grid was calculated using the field-based 3D-QSAR. The force-field-based QSAR model is an alignment-dependent method in which the interaction energy terms of the molecular field are correlated with biological activities using multivariate statistical analysis. In the 3D-QSAR model based on Gaussian force fields, interaction energy calculations were performed using steric, electrostatic, hydrogen bond donor (HBD), and hydrogen bond acceptor (HBA) potential fields using Gaussian equations for the field calculations. The fields were calculated on an orthohedral grid enclosing the training set molecules, with a spacing of 1 Å and extending 3 Å beyond the boundaries of this set. The threshold for van der Waals and electrostatic interactions was set at 30 kcal/mol, eliminating points closer than 2 Å from any of the atoms in the training set. During the PLS procedure, all variables (grid points) with a standard deviation less than 0.05 were removed.

The lattice and probe step sizes were adjusted automatically. The partial least squares (PLS) analysis is applied to construct the best model through the linear correlation of FFQSAR and GFQSAR concerning pEC_50_ [18,22,23]. The maximum number of PLS factors was set to 5. A cross-validation analysis was performed using the leave-one-out method.

The external predictive ability of each model constructed was assessed by calculating the predictive correlation coefficient (R^2^_test_). In addition, the models were also subjected to external validation criteria according to the test proposed by Golbraikh and Tropsha (see Supplementary Material in Section S2.4) [39,40]. All these calculations were carried out with the DTC Lab software tools (https://dtclab.webs.com/software-tools, accessed on 25 March 2021).

### 3.4. Prediction ADMET Properties

Drug candidates need to have good ADMET (absorption, distribution, metabolism, excretion, and toxicity) and drug-likeness profiles to initially estimate pharmacokinetic and drug-likeness parameters in the drug discovery process [41].

In this work, new candidates with ADMET properties include human intestinal absorption, steady-state volume of distribution (VDss), hepatic metabolism, total clearance, AMES toxicity, hepatotoxicity, and skin sensitization properties. ADMET can be predicted using pkCSM [29].

The prediction of drug similarity of new molecules is estimated using parameters based on Lipinski, Ghose, Veber, and Egan rules and their synthetic accessibility by applying the SwissADME web tool [31] (http://www.swissadme.ch, accessed on 25 March 2021). The SwissADME synthetic accessibility score is mainly based on the assumption of the molecular fragments in the “actually” obtainable molecules, which correlates with the ease of synthesis. The score is normalized to range from 1 (very easy) to 10 (very difficult to synthesise).

## 4. Conclusions

The structures included in this study have a reasonable structure–activity relationship and good correlation. The force and Gaussian-field models were generated and showed good R^2^ and Q^2^_LOO_ values for the models. The field-based model has R^2^ = 0.821 and Q^2^ = 0.804 based on the steric and electrostatic fields. The Gaussian model has R^2^ = 0.898 and Q^2^ = 0.886 based on the four field intensities of steric, electrostatic, hydrogen-bond acceptor (HBA), and hydrogen-bond donor (HBD). The analysis of both 3D-QSAR models indicates that the largest contributions are provided by steric and hydrogen bond acceptors properties (0.420 and 0.304, respectively). The models developed herein can be further applied to design new compounds with potent 5-HT_4_ receptor partial agonists. Finally, we found three factors that could effectively enhance the activity of 5-HT_4_R partial agonists:

(1) The four-carbon atom distance between the amide nitrogen and the aliphatic amine corresponding to fragment 3.
(2) Structural variability in fragment 3 considering aliphatic rings that provide a favourable hydrophobic source for activity
(3) The hydrogen bond acceptor groups in fragment 3 can enhance the activity of compounds.

**Figure 9:**
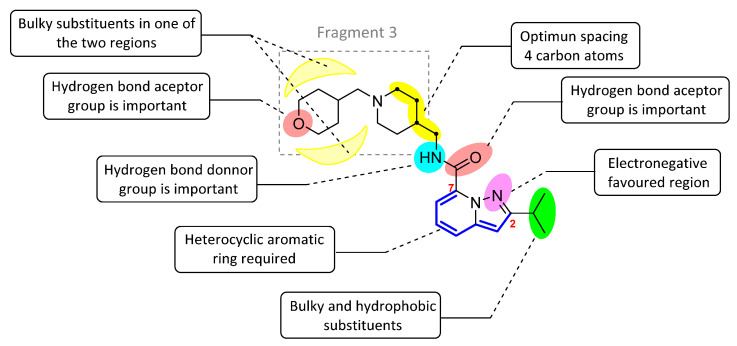
Main structure-activity relationships derived from this study.

The structural elements related to the biological activity of these compounds studied are shown in Figure 9.

Finally, the combination of the three factors showed better predicted biological activity than the single or the two factors (**var 8**). According to these rules, thirty-nine new molecules were designed, and the constructed 3D-QSAR models were used to predict the pEC_50_ value of the newly designed molecules. Ten new 5-HT_4_R partial agonists were selected as having promised biological activity compared to the studied compounds. Furthermore, the results of in silico studies suggested that these new 5-HT_4_R partial agonists have reasonable ADMET properties and drug-likeness. These results establish a theoretical basis for further study of these compounds. A deeper study focused on synthesizing of these compounds and the experimental study of their biological activity will pursue in future research.

## Figures and Tables

**Table 1:** Summary of statistical results from force field quantitative structure-activity relationship (FFQSAR) and field contributions.

						Fraction of Fields Included in the Model	
Fields	SD	R^2^_training_	R^2^_Scramble_	R^2^_test_	Stability	Steric	Electrostatic
S	0.654	0.719	0.554	0.329	0.229	1	
E	0.633	0.737	0.230	0.314	0.614		1
All	0.522	0.821	0.188	0.667	0.120	0.574	0.426

SD: Standard deviation of the regression. R^2^_training_ is the value for the regression (the coefficient of determination) of the training set. R^2^_scramble_ is the average value of R^2^ from a series of models built using scrambled activities; this value measures the degree to which the molecular fields can fit meaningless data. The value of R^2^_test_ for the predicted activities on the test set. Stability of the model predictions to changes in the training set composition. The steric (S) and electrostatic (E) field contributions in each model.

**Table 2:** Summary of statistical results from Gaussian field quantitative structure-activity relationship (GFQSAR) and field contributions.

						Field Contributions				
Fields	SD	R^2^_training_	R^2^_Scramble_	R^2^_test_	Stability	S	E	H	A	D
S	0.742	0.639	0.681	0.229	0.507	1.000				
E	0.362	0.914	0.769	0.573	0.688		1.000			
H	0.498	0.837	0.804	0.460	0.341			1.000		
A	0.567	0.789	0.645	0.548	0.587				1.000	
D	0.810	0.570	0.382	0.178	0.362					1.000
SE	0.563	0.792	0.765	0.557	0.017	0.708	0.293			
SH	0.565	0.791	0.784	0.405	0.019	0.509		0.491		
SA	0.507	0.831	0.790	0.319	0.253	0.534			0.466	
SD	0.572	0.785	0.763	0.657	0.226	0.675				0.325
EH	0.343	0.923	0.862	0.577	0.431		0.304	0.696		
EA	0.479	0.849	0.743	0.656	0.593		0.345		0.655	
ED	0.431	0.878	0.702	0.548	0.475		0.609			0.391
HA	0.387	0.902	0.835	0.615	0.453			0.561	0.440	
HD	0.473	0.853	0.824	0.405	0.217			0.734		0.266
AD	0.504	0.833	0.681	0.526	0.328				0.710	0.290
SEH	0.483	0.847	0.817	0.560	0.141	0.417	0.170	0.413		
SHA	0.443	0.871	0.824	0.628	0.225	0.356		0.326	0.318	
SHD	0.498	0.838	0.817	0.422	0.017	0.416	0.375		0.210	
SEA	0.467	0.857	0.807	0.681	0.250	0.466	0.147		0.387	
SED	0.498	0.837	0.799	0.492	0.027	0.555	0.194			0.251
SAD	0.486	0.845	0.812	0.617	0.148	0.470			0.356	0.175
EHA	0.332	0.928	0.846	0.604	0.454		0.171	0.481	0.348	
EHD	0.368	0.911	0.846	0.627	0.344		0.230	0.576		0.194
EAD	0.455	0.864	0.739	0.611	0.447		0.275		0.504	0.221
HAD	0.377	0.907	0.844	0.601	0.346			0.490	0.366	0.145
SEHA	0.410	0.890	0.834	0.670	0.257	0.321	0.109	0.295	0.275	
SEHD	0.443	0.871	0.836	0.556	0.099	0.365	0.131	0.331		0.172
SEAD	0.442	0.898	0.826	0.695	0.172	0.420	0.125		0.304	0.151
SHAD	0.430	0.879	0.840	0.644	0.186	0.328		0.281	0.260	0.132
EHAD	0.356	0.917	0.850	0.639	0.396		0.145	0.426	0.305	0.125
SEHAD	0.395	0.898	0.847	0.674	0.213	0.302	0.095	0.258	0.228	0.117

SD is the standard deviation of the regression. R^2^_training_ is the value for the regression (the coefficient of determination) of the training set. R^2^_scramble_ is the average value of R^2^ from a series of models built using scrambled activities; this value measures the degree to which the molecular fields can fit meaningless data. The value of R^2^_test_ for the predicted activities on the test set. Stability of the model predictions to changes in the training set composition. The steric (**S**), electronic (**E**), hydrophobic (**H**), hydrogen-bond donor (**D**), and hydrogen-bond acceptor (**A**) field contributions in each model.

**Table 3:** Experimental and calculated pEC_50_ and residual values for the analyzed compounds obtained with the force-field QSAR (FFQSAR) and Gaussian-field QSAR (GFQSAR) model. The highlighted rows show the test set compounds.

		FFQSAR		GFQSAR				FFQSAR		GFQSAR	
Comp.	pEC_50_exp_	pEC_50_calc_	Res.	pEC_50_calc_	Res.	Comp.	pEC_50_exp_	pEC_50_calc_	Res.	pEC_50_calc_	Res.
**1**	8.921	8.996	−0.075	8.404	0.518	**32**	7.246	6.825	0.421	6.993	0.253
**2**	8.585	8.836	−0.251	9.127	−0.542	**33**	6.842	7.797	−0.955	8.065	−1.223
**3**	7.398	8.416	−1.018	7.211	0.187	**34**	6.315	6.676	−0.361	6.240	0.076
**4**	7.509	7.745	−0.236	7.716	−0.207	**35**	7.268	6.893	0.375	7.441	−0.173
**5**	5.648	6.033	−0.385	5.894	−0.246	**36**	6.942	6.924	0.018	7.180	−0.238
**6**	6.284	6.418	−0.134	6.683	−0.399	**37**	8.310	8.321	−0.011	8.586	−0.276
**7**	6.331	5.984	0.347	6.177	0.154	**38**	9.523	9.551	−0.028	9.631	−0.108
**8**	6.133	6.173	−0.040	6.098	0.035	**39**	8.060	7.735	0.325	7.305	0.755
**9**	8.699	8.736	−0.037	8.733	−0.034	**40**	8.076	8.292	−0.216	7.911	0.165
**10**	7.141	8.076	−0.935	7.222	−0.081	**41**	7.102	8.080	−0.978	8.083	−0.981
**11**	7.703	7.675	0.028	7.967	−0.264	**42**	6.223	5.805	0.418	5.591	0.632
**12**	6.067	6.801	−0.734	6.559	−0.492	**43**	5.712	6.893	−1.181	6.000	−0.288
**13**	8.244	7.459	0.785	8.088	0.156	**44**	7.983	7.459	0.524	7.512	0.471
**14**	8.000	7.932	0.068	8.196	−0.196	**45**	5.712	5.226	0.486	6.499	−0.787
**15**	8.009	7.568	0.441	7.775	0.234	**46**	8.824	9.078	−0.254	9.024	−0.200
**16**	8.824	8.294	0.530	8.722	0.102	**47**	9.155	8.822	0.333	8.597	0.558
**17**	8.469	7.331	1.138	7.541	0.928	**48**	7.208	7.169	0.039	6.673	0.535
**18**	9.301	8.681	0.620	9.184	0.117	**49**	7.009	6.466	0.543	7.435	−0.426
**19**	6.301	6.646	−0.345	5.982	0.319	**50**	6.120	6.400	−0.280	6.303	−0.183
**20**	5.867	6.333	−0.466	6.292	−0.425	**51**	9.222	9.270	−0.048	9.368	−0.146
**21**	7.658	7.808	−0.150	7.641	0.017	**52**	7.866	7.670	0.196	7.525	0.341
**22**	7.237	7.235	0.002	7.383	−0.146	**53**	10.000	8.778	1.222	8.970	1.030
**23**	7.469	8.233	−0.764	7.944	−0.475	**54**	8.046	8.168	−0.122	7.983	0.063
**24**	7.745	7.490	0.255	7.766	−0.021	**55**	6.099	6.030	0.069	5.722	0.377
**25**	7.738	7.225	0.513	7.848	−0.110	**56**	8.056	7.701	0.355	8.100	−0.044
**26**	7.409	7.349	0.060	7.366	0.043	**57**	8.886	8.488	0.398	9.006	−0.120
**27**	7.301	7.367	−0.066	7.527	−0.226	**58**	9.523	8.838	0.685	8.928	0.595
**28**	7.959	8.241	−0.282	7.843	0.116	**59**	9.398	9.487	−0.089	9.815	−0.417
**29**	6.076	6.727	−0.651	6.162	−0.086	**60**	5.963	6.700	−0.737	6.536	−0.573
**30**	8.319	7.986	0.333	7.491	0.829	**61**	7.678	6.177	1.501	6.403	1.275
**31**	8.284	8.662	−0.378	8.659	−0.375	**62**	7.377	7.497	−0.120	8.061	−0.684

Res.: Residual value. Comp.: Compound number

**Table 4:** The proposed structures of new derivates and their predicted pEC_50_ values using the FFQSAR and GFQSAR models.

ID	Structures	FFQSAR	GFQSAR	ID	Structures	FFQSAR	GFQSAR
**var1**	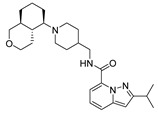	8.283	9.221	**var6**	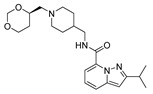	8.698	9.111
**var2**	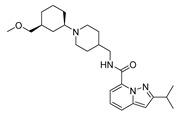	8.209	9.375	**var7**	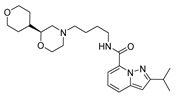	8.690	9.547
**var3**	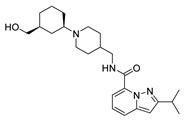	8.285	9.432	**var8**	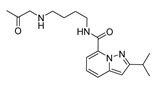	9.417	9.259
**var4**	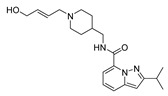	8.719	9.114	**var9**	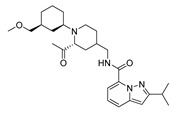	8.818	9.700
**var5**	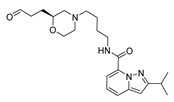	8.775	9.513	**var10**	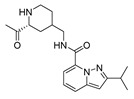	8.641	9.849

**Table 5:** Absorption, distribution, metabolism, excretion, and toxicity (ADMET) properties of new designed molecules.

	Absorption	Distribution	Metabolism							Excretion	Toxicity		
			Substrate		Inhibitor								
ID	IA ^1^	VDss ^2^	2D6	3A4	1A2	2C19	2C9	2D6	3A4	TC ^3^	AMES	Hepatotoxicity	Skin Sensitization
**53**	92.738	1.473	Yes	Yes	No	No	No	Yes	Yes	0.825	No	Yes	No
**var1**	95.344	1.158	No	Yes	No	No	No	No	Yes	0.626	No	Yes	No
**var2**	95.054	1.152	No	Yes	No	No	No	No	No	0.776	No	Yes	No
**var3**	94.483	1.084	No	Yes	No	No	No	No	No	0.789	No	Yes	No
**var4**	95.045	0.941	No	Yes	No	No	No	No	No	0.837	No	Yes	No
**var5**	95.572	0.826	No	Yes	No	No	No	No	No	1.258	No	Yes	No
**var6**	96.306	0.84	No	Yes	No	No	No	No	No	0.936	No	Yes	No
**var7**	95.844	0.952	No	Yes	No	No	No	No	No	1.06	No	Yes	No
**var8**	94.815	0.584	No	No	No	No	No	No	No	1.171	No	Yes	No
**var9**	92.905	0.961	No	No	No	No	No	No	Yes	0.719	No	Yes	No
**var10**	95.300	0.671	No	No	No	No	No	No	Yes	0.881	No	Yes	No

^1^ IA is intestinal absorption, values expressed in % absorption. ^2^ VDss is volume of distribution, values expressed in log L kg^−1^. ^3^ TC is total clearance, values expressed in log mL min^−1^ kg^−1^.

**Table 6:** Drug likeness of novel designed molecules based on Lipinski, Ghose, Veber, and Egan rules, and their synthetic accessibility.

ID	Lipinski	Ghose	Veber	Egan	Synthetic Accessibility
**53**	Yes	Yes	Yes	Yes	3.26
**var1**	Yes	No	Yes	Yes	4.77
**var2**	Yes	Yes	Yes	Yes	4.55
**var3**	Yes	Yes	Yes	Yes	4.4
**var4**	Yes	Yes	Yes	Yes	3.52
**var5**	Yes	Yes	No	Yes	3.68
**var6**	Yes	Yes	Yes	Yes	4.07
**var7**	Yes	Yes	Yes	Yes	3.97
**var8**	Yes	Yes	Yes	Yes	2.71
**var9**	Yes	No	Yes	Yes	4.89
**var10**	Yes	Yes	Yes	Yes	3.48

## Data Availability

The data presented in this study are available in Supplementary Material or on request from the corresponding author.

## Supplementary Materials

The following are available online at https://www.mdpi.com/article/10.3390/ijms22073602/s1.

## Abbreviations

5-HT_4_R	Receptor 5-HT_4_
AD	Alzheimer’s disease
S	Steric
E	Electrostatic
H	Hydrophobic
HBA	Hydrogen bond acceptor
HBD	Hydrogen bond donor
ADMET	Absorption, distribution, metabolism, excretion, and toxicity
FFQSAR	Force-field based QSAR
GFQSAR	Gaussian-field based QSAR
CCC	Correlation coefficient of concordance
3D-QSAR	Three-dimensional quantitative structure–activity relationship
