# Supplementary material for: Understanding the Molecular Basis of 5-HT4 Receptor Partial Agonists through 3D-QSAR Studies
Source: Int J Mol Sci. 2021 Mar 30;22(7):3602. doi: 10.3390/ijms22073602 (PMC8036435; doi:10.3390/ijms22073602)
Supplement: Supplementary file 1 [file ijms-22-03602-s001.pdf]

Supplementary Material

Understanding the Molecular Basis of 5-HT_4 Receptor Partial Agonists through 3D-QSAR Studies

Alejandro Castro-Alvarez [1,*], Emigdio Chávez-Ángel [2] and Ronald Nelson [3,*]

[1] Laboratorio de Bioproductos Farmacéuticos y Cosméticos, Centro de Excelencia en Medicina Traslacional, Facultad de Medicina, Universidad de La Frontera, Av. Francisco Salazar 01145, Temuco 4780000, Chile.; alejandro.castro.a@ufrontera.cl
[2] Catalan Institute of Nanoscience and Nanotechnology (ICN2), CSIC and BIST, Campus UAB, Bellaterra, 08193 Barcelona, Spain emigdio.chavez@icn2.cat
[3] Departamento de Química, Facultad de Ciencias, Universidad Católica del Norte, Av. Angamos 0610, Antofagasta, Chile; rnelson@ucn.cl
* Correspondence: alejandro.castro.a@ufrontera.cl (A.C.-A.); rnelson@ucn.cl (R.N.)

Contents

S1. EC_{50} experimental 2
Table S1. Biological activity of all compounds. The highlighted rows show the test set compounds. 2
S2. Details of the Compounds 2
S2.1. Dataset Collection 2
S2.2. Alignment 3
S2.3. Field-Based QSAR Model 3
S2.4. Validation of the 3D-QSAR Model 3
S2.5. Prediction ADMET Properties 7
S3. Novel derivatives 8
Table S3. 39 new compounds with predicted pEC_{50}. 8
Table S4. Structural characteristics of the 10 compounds selected with SwissADME. 9
Table S5. Lipophilicity calculated for the 10 compounds selected with SwissADME. 10
Table S6. Solubility calculated for the 10 compounds selected with SwissADME. 10
S4. References 11

S1. EC_{50} experimental

Table S1. *Biological activity of all compounds. The highlighted rows show the test set compounds.*

Compound	EC_{50} nM	pEC_{50}	Compound	EC_{50} nM	pEC_{50}
**1**	1.199	8.921	**32**	56.754	7.246
**2**	2.600	8.585	**33**	143.880	6.842
**3**	39.994	7.398	**34**	484.172	6.315
**4**	30.974	7.509	**35**	53.951	7.268
**5**	2249.055	5.648	**36**	114.288	6.942
**6**	519.996	6.284	**37**	4.898	8.310
**7**	466.659	6.331	**38**	0.300	9.523
**8**	736.207	6.133	**39**	8.710	8.060
**9**	2.000	8.699	**40**	8.395	8.076
**10**	72.277	7.141	**41**	79.068	7.102
**11**	19.815	7.703	**42**	598.412	6.223
**12**	857.038	6.067	**43**	1940.886	5.712
**13**	5.702	8.244	**44**	10.399	7.983
**14**	10.000	8.000	**45**	1940.886	5.712
**15**	9.795	8.009	**46**	1.500	8.824
**16**	1.500	8.824	**47**	0.700	9.155
**17**	3.396	8.469	**48**	61.944	7.208
**18**	0.500	9.301	**49**	97.949	7.009
**19**	500.035	6.301	**50**	758.578	6.120
**20**	1358.313	5.867	**51**	0.600	9.222
**21**	21.979	7.658	**52**	13.614	7.866
**22**	57.943	7.237	**53**	0.100	10.000
**23**	33.963	7.469	**54**	8.995	8.046
**24**	17.989	7.745	**55**	796.159	6.099
**25**	18.281	7.738	**56**	8.790	8.056
**26**	38.994	7.409	**57**	1.300	8.886
**27**	50.003	7.301	**58**	0.300	9.523
**28**	10.990	7.959	**59**	0.400	9.398
**29**	839.460	6.076	**60**	1088.930	5.963
**30**	4.797	8.319	**61**	20.989	7.678
**31**	5.200	8.284	**62**	41.976	7.377

S2. Details of the Compounds

S2.1. Dataset Collection

A total of 62 partial agonists of the 5-HT_4 receptor which showed promissory potency were collected from the literature [1–3]. All the compounds with pEC_{50} values ranging from 5.64 to 10.0 were used in this study. The geometry for all these molecules was converted into a 3D structure using OCHEM. The 3D structure of the molecules was processed with OMEGA [4] module using the following parameters: (i) AM1_BCC Force field, (ii) FixpKa from the QUAPAC package for all possible

ionisation states at a given biological pH, (iii) one low energy conformation per ligand. Force- and Gaussian-field 3D-QSAR calculations were performed for all the molecules. All the training and test set molecules with experimental and predicted EC_{50} values were listed in Table 1.

S2.2. Alignment

Alignment of molecules is the most crucial input for the generation of 3D-QSAR models. The compound with the highest activity (**53**) was used as the template molecule. A shape-based alignment was used for all conformers of each ligand. These alignments were carried out with ROCS suite [5]. Finally, each ligand's best conformer was filtered considering electrostatic field compound **53,** as is shown in Figure S1.

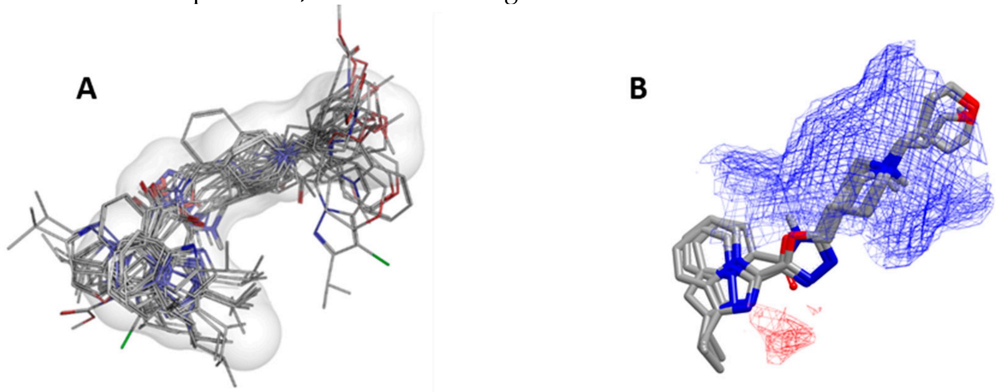

Figure S1. (**A**) structure aligned with ROCS. (**B**) structures aligned considering the electrostatic field.

S2.3. Field-Based QSAR Model

3D-QSAR analysis using Field-based methods was performed by QSAR tool of Schrodinger Suite. The 3D-QSAR method constructs the model by relating the known activities and molecular elements of a set of aligned compounds. The steric and electrostatic field around the ligand in a 3D-grid was calculated using field-based 3D-QSAR. Force-field based QSAR model (henceforward FFQSAR) is an alignment-dependent method in which molecular field interaction energy terms are correlated with biological activities/responses using multivariate statistical analyses. In Gaussian-field, 3D-QSAR model interaction energy calculations were performed using steric, electrostatic, hydrogen bond donor (HBD), and hydrogen bond acceptor (HBA) potential fields and it uses Gaussian equations for field calculations (henceforward GFQSAR).

The lattice and probe step sizes were adjusted automatically. The partial least squares (PLS) analysis is applied to construct the best model through the linear correlation of FFQSAR and GFQSAR concerning pEC_{50} [1–3]. A cross-validation analysis was performed using the leave-one-out method. Finally, the optimum number of components was identified by the cross-validation method. Correlation and cross-validation coefficients (Q^2 and R^2 respectively) were calculated according to the formula:

$$Q^2 = 1 - \frac{\sum_{i=1}^{\infty}(\hat{y}_i - y_i)^2}{\sum_{i=1}^{\infty}(y_i - \bar{y})^2} \quad (1)$$

$$R^2 = \frac{[\sum(y_i - \bar{y}_i)(\hat{y}_i - \hat{y})]^2}{\sum(y_i - \bar{y}_i)^2 \times \sum(\hat{y}_i - \hat{y})^2} \quad (2)$$

where $\hat{y}_i$ and y_i are predicted, observed activity values, and $\bar{y}$ and $\hat{y}$ are observed and predicted mean activity values of the training set, respectively. The $\sum_{i=1}^{\infty}(y_i - \bar{y})^2$ is the predictive residual sum of squares (PRESS).

High Q^2 and R^2 ($Q^2 > 0.6$, $R^2 > 0.8$) values are regarded as proof of the built model's high predictive ability.

S2.4. Validation of the 3D-QSAR Model

A good internal validation showed only a high Q^2 in the training set of compounds, but it did not indicate the established models' high predictive ability. Therefore, external validation was indispensable. The predictive power of 3D-

QSAR models was validated by calculating biological activities of the compounds which were not included in the training set and used as a test set.

The predictive correlation coefficient R^2_{test} ($R^2_{test} > 0.6$) [6], based on the test set was calculated using Equation (3):

$$R^2_{test} = \left(\frac{SD - PRESS}{SD}\right) \quad (3)$$

The sum of squared deviation (SD) between the biological activities of the test set molecules and the mean activity of the training set molecules. $PRESS$ is the sum of squared derivations between the predicted and actual activities of the test set molecules.

The performance of the regression models constructed here was evaluated using the root mean squared error ($RMSE$), mean absolute error (MAE) ($RMSE$ and MAE close to zero), residual sum of squares (RSS) and concordance correlation coefficient (CCC; where $CCC \geq 0.85$) of the training and validation sets [7]. The RMSE and the MAE are calculated for the data set as Equations (4) to (7):

$$RMSD = \sqrt{\frac{\sum_{i=1}^{n}(y_i - \hat{y}_i)^2}{n}} \quad (4)$$

$$MAE = \frac{\sum_{i=1}^{n}|y_i - \hat{y}_i|}{n} \quad (5)$$

$$RSS = \sum_{i=1}^{n}(y_i - \hat{y}_i)^2 \quad (6)$$

$$CCC = \frac{2\sum_{i=1}^{n}(y_i - \bar{y})(\hat{y}_i - \hat{y})}{\sum_{i=1}^{n}(y_i - \bar{y})^2 + \sum_{i=1}^{n}(\hat{y}_i - \hat{y})^2 + n(\bar{y} - \hat{y})^2} \quad (7)$$

To obtain the best predictive model for the test set, additional validation of model, the following:

$$r_0^2 = 1 - \frac{\sum(y_i - k \times \hat{y}_i)^2}{\sum(y_i - \bar{y})^2} \quad (8)$$

$$r_0'^2 = 1 - \frac{\sum(\hat{y}_i - k \times y_i)^2}{\sum(\hat{y}_i - \hat{y}_i)^2} \quad (9)$$

$$k = \frac{\sum(y_i \times \hat{y}_i)}{\sum(\hat{y}_i)^2} \quad (10)$$

$$k' = \frac{\sum(y_i \times \hat{y}_i)}{\sum(y_i)^2} \quad (11)$$

$$\frac{(r^2 - r_0^2)}{r^2} < 0.1 \;\; or \;\; \frac{(r^2 - r_0'^2)}{r^2} < 0.1 \quad (12)$$

$0.85 \leq k \leq 1.15$ or $0.85 \leq k' \leq 1.15$ r_0^2 and $r_0'^2$ are squared correlation coefficients of determination for regression lines through the origin between predicted (y) and observed (x) activities and vice versa. The values of k and k_0 are the slopes of their models, respectively.

To further assess the models, another statistical validation parameter r_m^2 and Δr_m^2 were determined by the following Equations (13) and (14):

$$r_m^2 = r^2\left(1 - \sqrt{|r^2 - r_0^2|}\right) \quad (13)$$

$$\Delta r_m^2 = |r_m^2 - r_m'^2| \quad (14)$$

r_m^2 value of more than 0.5 ($r_m^2 > 0.5$) and $\Delta r_m^2 < 0.2$ show good external predictability of the models.

The internal and external validation for GFQSAR meet threshold values which demonstrates reliability of the model with the SEAD fields. In contrast, FFQSAR has the concordance correlation coefficient (CCC) value below the tolerated

threshold value (FFQSAR is 0.815 and the threshold > 0.85). Therefore, the design of new analogues will be based on the GFQSAR model.

Table S2. *Summary of results obtained using Force- and Gaussian-field.*

Internal validation parameters

Parameters	Threshold Value	Force-field QSAR	Gaussian-field QSAR
$R^2_{training}$	> 0.8	0.821	0.898
Q^2_{LOO}	> 0.6	0.804	0.886
F		188.056	360.131
P		3.31E-13	2.92E-14

External validation parameters

Parameters	Threshold Value	Force-field QSAR	Gaussian-field QSAR
R^2_{test}	> 0.6	0.667	0.695
r^2_0	Close to value of R^2_{test}	0.574	0.678
r'^2_0	Close to value of R^2_{test}	0.661	0.656
k	0.85 < k < 1.15	0.996	0.992
k'	0.85 < k' < 1.15	0.997	1.003
$(r^2 - r^2_0)/r^2$	< 0.1	0.139	0.025
$(r^2 - r'^2_0)/r^2$	< 0.1	0.008	0.056
$\lvert r^2_0 - r'^2_0 \rvert$	< 0.3	0.087	0.021
r^2_m	> 0.5	0.558	0.841
Q^2F_1	> 0.6	0.785	0.819
Q^2F_2	> 0.6	0.614	0.674
CCC	> 0.85	0.815	0.851
Δr^2_m	< 0.2	0.065	0.077

Error-based metrics:

Parameters	Force-field QSAR	Gaussian-field QSAR
RMSEP	0.632	0.581
SD	0.431	0.377
MAE	0.473	0.450

Q^2 = the square of the LOO cross-validation (CV) coefficient; R^2_{test} is the regression coefficient for the test set exclusively; "r^2_0" and "k" are the correlation coefficient between the actual and predicted activities for test set and the respective slope of regression; and "$r_0'^2$" and "k'" are the correlation coefficient between the predicted and actual activities for test set and the respective slope of regression. "r^2_m" was defined in equation 11. Parameters are defined in the section "Validation of the QSAR model".

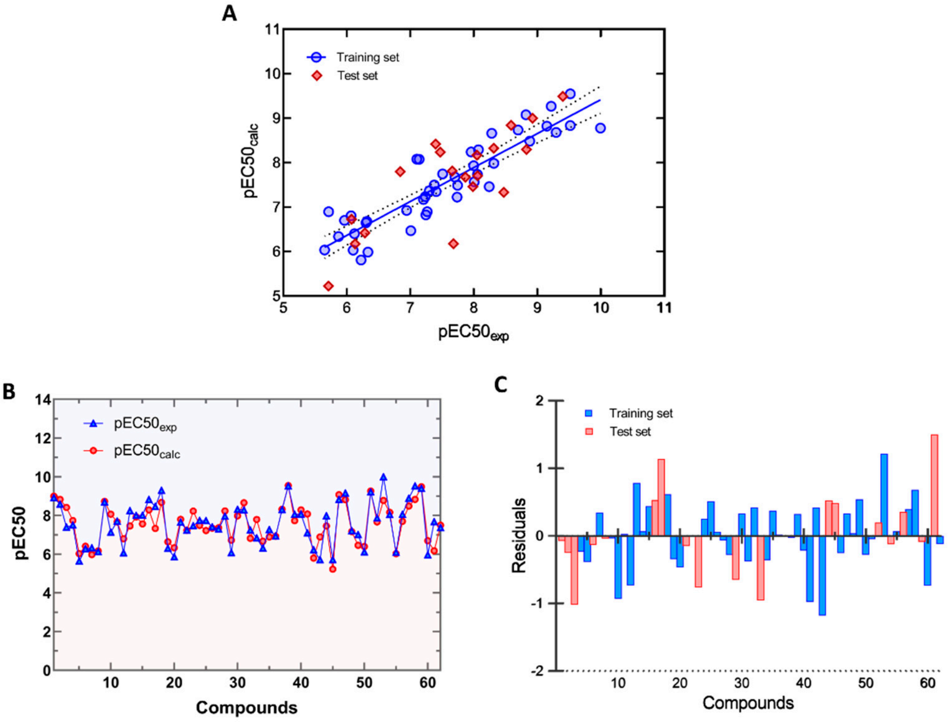

Figure S2. (**A**) Plots of experimental versus calculated pEC50 values for the training and test set molecules for Force field QSAR (FFQSAR) model. (**B**) Residual plots between predicted and experimental values for FFQSAR. (**C**) FFQSAR predictions for every molecule in the complete set

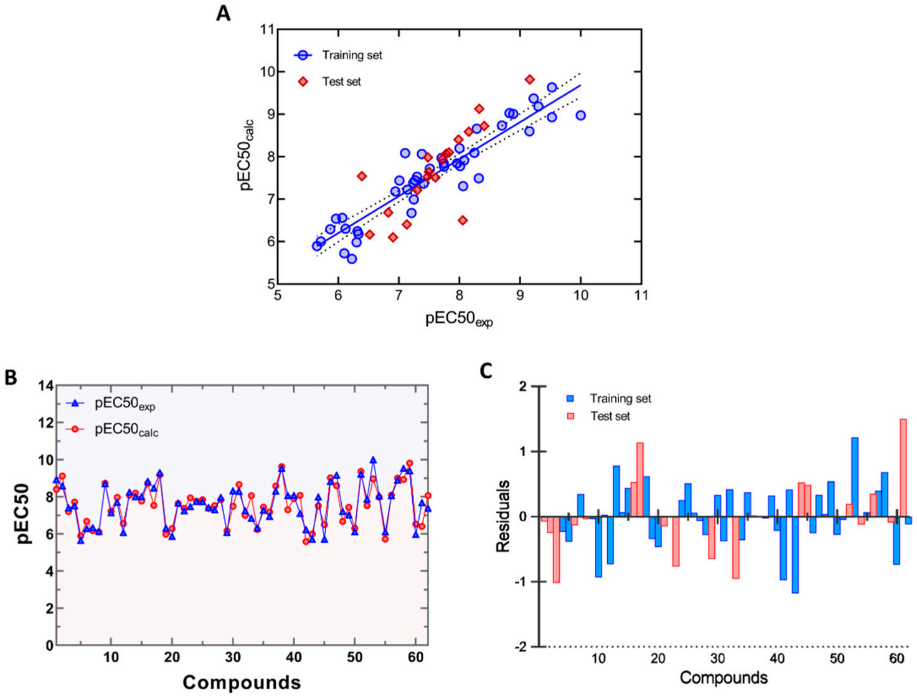

Figure S3. (**A**) Plots of experimental versus calculated pEC50 values for the training and test set molecules for Gaussian field 3D-QSAR (GFQSAR) model. (**B**) Residual plots between predicted and observed values for GFQSAR. (**C**) GFQSAR predictions for every molecule in the complete set

S2.5. Prediction ADMET Properties

Drug candidates need to have good ADMET (Absorption, Distribution, Metabolism, Excretion and Toxicity) and drug-likeness profiles to initially estimate pharmacokinetic and drug-likeness parameters in the drug discovery process [8].

In this work, new candidates with ADMET properties include human intestinal absorption, steady-state volume of distribution (VDss), hepatic metabolism, total clearance, AMES toxicity, and hepatotoxicity and skin sensitisation properties. ADMET can be predicted using pkCSM [9].

The prediction of drug similarity of new molecules is estimated using parameters based on Lipinski, Ghose, Veber and Egan rules, and their synthetic accessibility by applying the SwissADME web tool [10] (http://www.swissadme.ch). The SwissADME synthetic accessibility score is mainly based on the assumption of the molecular fragments in the "actually" obtainable molecules which correlates with the ease of synthesis. The score is normalised to range from 1 (very easy) to 10 (very difficult to synthesise).

S3. Novel derivatives

Table S3. *39 new compounds with predicted pEC₅₀.*

Num	SMILES	Selected	GFQSAR	FFQSAR
1	CC(C)c(c1)nn(c12)c(ccc2)C(=O)NC[C@H]3CC[N@@H+](CC3)CC4CCOCC4		8.985	8.714
2	CC(C)c(c1)nn(c12)c(ccc2)C(=O)NC[C@H]3CC[N@@H+](CC3)[C@@H]4CCC[C@@H]([C@H]45)COCC5	**var1**	9.221	8.283
3	CC(C)c(c1)nn(c12)c(ccc2)C(=O)NC[C@H]3CC[N@@H+](CC3)[C@H](CCC4)C[C@H]4COC	**var2**	9.375	8.209
4	CC(C)c(c1)nn(c12)c(ccc2)C(=O)NC[C@H]3CC[N@@H+](CC3)[C@H](CCC4)C[C@H]4CO	**var3**	9.432	8.285
5	CC(C)c(c1)oc(c12)c(ccc2)C(=O)NC[C@H]3CC[N@@H+](CC3)CC4CCOCC4		8.963	8.411
6	CC(C)c(c1)oc(c12)c(ccc2)C(=O)NC[C@H]3CC[N@@H+](CC3)CCCCOC		9.085	8.403
7	CC(C)c(c1)oc(c12)c(ccc2)C(=O)NC[C@H]3CC[N@@H+](CC3)C(C)(C)CCCO		9.061	8.017
8	CC(C)c(c1)oc(c12)c(ccc2)C(=O)NC[C@H]3CC[N@@H+](CC3)C(C)(C)C4CCOCC4		8.993	8.049
9	CC(C)c(c1)oc(c12)c(ccc2)C(=O)NC[C@H]3CC[N@@H+](CC3)CCCCO		9.031	8.379
10	CC(C)c(c1)oc(c12)c(ccc2)C(=O)NC[C@H]3CC[N@@H+](CC3)C/C=C/CO		9.041	8.418
11	CC(C)c(c1)sc(c12)c(ccc2)C(=O)NC[C@H]3CC[N@@H+](CC3)CC4CCOCC4		8.533	8.272
12	CC(C)(C)c(c1)oc(c12)c(ccc2)C(=O)NC[C@H]3CC[N@@H+](CC3)CC4CCOCC4		8.794	7.620
13	CC(C)c(c1)nn(c12)c(ccc2)C(=O)NC[C@H]3CC[N@@H+](CC3)[C@@H](C4)CC[C@H]([C@@H]45)CCC[C@H]5OC		9.179	8.077
14	CC(C)c(c1)nn(c12)c(ccc2)C(=O)NC[C@H]3CC[N@@H+](CC3)C/C=C/CO	**var4**	9.114	8.719
15	CC(C)c(c1)nn(c12)c(ccc2)C(=O)NC[C@H]3CC[N@@H+](CC3)C[C@H](O)CC		9.090	8.659
16	CC(C)c(c1)nn(c12)c(ccc2)C(=O)NC[C@H]3CC[N@@H+](CC3)[C@H](C4=O)CCCC4		8.821	8.363
17	CCC(=O)C[N@H+](CC1)CC[C@@H]1CNC(=O)c(ccc2)n(c23)nc(c3)C(C)C		8.779	8.882
18	CC(C)c(c1)nn(c12)c(ccc2)C(=O)NC[C@H]3CC[N@@H+](CC3)C[C@H]4CCCCO4		8.998	8.698
19	CC(C)c(c1)nn(c12)c(ccc2)C(=O)NC[C@H]3CC[N@@H+](CC3)C[C@H]4CCCO4		8.992	8.710
20	CC(C)c(c1)nn(c12)c(ccc2)C(=O)NC[C@H]3CC[N@@H+](CC3)Cc4ccco4		8.802	8.853
21	CC(C)c(c1)nn(c12)c(ccc2)C(=O)NC[C@H]3CC[N@@H+](CC3)C[C@H](O)CC4CCCCC4		9.044	8.800
22	CC(C)c(c1)nn(c12)c(ccc2)C(=O)NC[C@H]3CC[N@@H+](CC3)C[C@H](O)C4CCCCC4		9.009	8.138
33	CC(C)c(c1)nn(c12)c(ccc2)C(=O)NCCCC[N@@H+](CCO3)C[C@@H]3CCC=O	**var5**	9.513	8.775
23	CC(C)c(c1)nn(c12)c(ccc2)C(=O)NC[C@H]3CC[N@@H+](CC3)C[C@H]4CCOCO4	**var6**	9.111	8.698
24	CC(C)c(c1)nn(c12)c(ccc2)C(=O)NC[C@H]3CC[N@@H+](CC3)[C@@H](N4)CCC[C@@H]4CN		9.001	8.475
25	CC(C)c(c1)nn(c12)c(ccc2)C(=O)NC[C@H]3CC[N@@H+](CC3)[C@@H](N4)CCC[C@@H]4CNC		9.104	8.529
26	CC(C)c(c1)nn(c12)c(ccc2)-c3cnc(o3)[C@H]4CC[N@@H+](CC4)[C@H](CCC5)C[C@H]5CN		6.995	6.582
27	CC(C)c(c1)nn(c12)c(ccc2)-c3cnc(o3)[C@H]4CC[N@@H+](CC4)[C@H](CCC5)C[C@H]5CO		7.223	6.966
28	CC(C)c(c1)nn(c12)c(ccc2)C(=O)NC[C@H]3CC[N@@H+](CC3)[C@@H](N4)CCC[C@@H]4CN		9.022	8.705
29	CC(C)c(c1)nn(c12)c(ccc2)C(=O)NC[C@H]3CC[N@@H+](CC3)[C@H](CCC4)C[C@H]4C(=O)OC		9.139	7.681
30	C1CCCCC1C(=O)[C@@H]2C[C@@H](CCC2)[N@H+](CC3)CC[C@@H]3CNC(=O)c(ccc4)n(c45)nc(c5)C(C)C		9.004	7.819
31	COC(=O)CCC[N@H+](CC1)CC[C@@H]1CNC(=O)c(ccc2)n(c23)nc(c3)C(C)C		8.820	9.203
32	CC(C)c(c1)nn(c12)c(ccc2)C(=O)NCCCC[N@@H+](CCO3)C[C@H]3C		9.203	8.012
34	CC(C)c(c1)nn(c12)c(ccc2)C(=O)NCCCCC3CCOCC3		9.347	6.535
35	CC(C)c(c1)nn(c12)c(ccc2)C(=O)NCCCC[N@@H+](CCO3)C[C@@H]3C4CCOCC4	**var7**	9.547	8.690
36	CC(=O)C[NH2+]CCCCNC(=O)c(ccc1)n(c12)nc(c2)C(C)C	**var8**	9.259	9.417
37	CC(C)c(c1)nn(c12)c(ccc2)C(=O)NC[C@H](C[C@@H]3C=O)CC[N@@H+]3[C@H](CCC4)C[C@H]4CO	**var9**	9.700	8.818
38	CC(=O)[C@H]1C[C@H](CC[NH2+]1)CNC(=O)c(ccc2)n(c23)nc(c3)C(C)C	**var10**	9.849	8.641
39	CC(C)c(c1)nn(c12)c(ccc2)-c3cn(nn3)[C@H]4CC[N@@H+](CC4)CC5CCOCC5		7.488	8.289

Table S4. *Structural characteristics of the 10 compounds selected with SwissADME.*

Molecule	SMILES	Formula	MW	Heavy atoms	Aromatic heavy atoms	Fraction Csp3	Rotatable bonds	H-bond acceptors	H-bond donors	MR	TPSA
**var1**	O=C(c1cccc2n1nc(c2)C(C)C)NC[C@@H]1CCN(CC1)[C@@H]1CCC[C@H]2[C@H]1CCOC2	C26H38N4O2	438.61	32	9	0.69	6	4	1	131.62	58.87
**var2**	COC[C@H]1CCC[C@H](C1)N1CC[C@H](CC1)CNC(=O)c1cccc2n1nc(c2)C(C)C	C25H38N4O2	426.59	31	9	0.68	8	4	1	128.93	58.87
**var3**	OC[C@H]1CCC[C@H](C1)N1CC[C@H](CC1)CNC(=O)c1cccc2n1nc(c2)C(C)C	C24H36N4O2	412.57	30	9	0.67	7	4	2	124.2	69.87
**var4**	OC/C=C/CN1CC[C@H](CC1)CNC(=O)c1cccc2n1nc(c2)C(C)C	C21H30N4O2	370.49	27	9	0.52	8	4	2	111.42	69.87
**var5**	O=CCC[C@@H]1OCCN(C1)CCCCNC(=O)c1cccc2n1nc(c2)C(C)C	C22H32N4O3	400.51	29	9	0.59	11	5	1	116.82	75.94
**var6**	O=C(c1cccc2n1nc(c2)C(C)C)NC[C@@H]1CCN(CC1)C[C@H]1CCOCO1	C22H32N4O3	400.51	29	9	0.64	7	5	1	115.59	68.1
**var7**	O=C(c1cccc2n1nc(c2)C(C)C)NCCCCN1CCO[C@H](C1)C1CCOCC1	C24H36N4O3	428.57	31	9	0.67	9	5	1	125.21	68.1
**var8**	CC(=O)CNCCCCNC(=O)c1cccc2n1nc(c2)C(C)C	C18H26N4O2	330.42	24	9	0.5	10	4	2	94.61	75.5
**var9**	COC[C@H]1CCC[C@H](C1)N1CC[C@@H](C[C@@H]1C(=O)C)CNC(=O)c1cccc2n1nc(c2)C(C)C	C27H40N4O3	468.63	34	9	0.67	9	5	1	138.74	75.94
**var10**	CC(=O)[C@@H]1NCC[C@@H](C1)CNC(=O)c1cccc2n1nc(c2)C(C)C	C19H26N4O2	342.44	25	9	0.53	6	4	2	101.22	75.5

Table S5. *Lipophilicity calculated for the 10 compounds selected with SwissADME.*

Molecule	Canonical SMILES	iLOGP	XLOGP3	WLOGP	MLOGP	Silicos-IT Log P	Consensus Log P
**var1**	O=C(c1cccc2n1nc(c2)C(C)C)NC[C@@H]1CCN(CC1)[C@@H]1CCC[C@H]2[C@H]1CCOC2	4.74	4.03	3.72	3.4	3.15	3.81
**var2**	COC[C@H]1CCC[C@H](C1)N1CC[C@H](CC1)CNC(=O)c1cccc2n1nc(c2)C(C)C	4.55	4	3.72	3.19	3.3	3.75
**var3**	OC[C@H]1CCC[C@H](C1)N1CC[C@H](CC1)CNC(=O)c1cccc2n1nc(c2)C(C)C	3.85	3.46	3.07	2.98	2.75	3.22
**var4**	OC/C=C/CN1CC[C@H](CC1)CNC(=O)c1cccc2n1nc(c2)C(C)C	3.8	2.24	2.07	2.26	2.46	2.57
**var5**	O=CCC[C@@H]1OCCN(C1)CCCCNC(=O)c1cccc2n1nc(c2)C(C)C	3.75	2.07	2.27	1.68	3.12	2.58
**var6**	O=C(c1cccc2n1nc(c2)C(C)C)NC[C@@H]1CCN(CC1)C[C@H]1CCOCO1	4	2.62	2.28	2.16	2.36	2.69
**var7**	O=C(c1cccc2n1nc(c2)C(C)C)NCCCCN1CCO[C@H](C1)C1CCOCC1	4.63	2.88	2.71	2.18	3.15	3.11
**var8**	CC(=O)CNCCCCNC(=O)c1cccc2n1nc(c2)C(C)C	2.92	1.87	2.15	1.58	2.57	2.22
**var9**	COC[C@H]1CCC[C@H](C1)N1CC[C@@H](C[C@@H]1C(=O)C)CNC(=O)c1cccc2n1nc(c2)C(C)C	4.17	3.78	3.68	2.72	3.37	3.54
**var10**	CC(=O)[C@@H]1NCC[C@@H](C1)CNC(=O)c1cccc2n1nc(c2)C(C)C	2.94	1.94	1.76	1.81	2.16	2.12

Table S6. *Solubility calculated for the 10 compounds selected with SwissADME.*

Molecule	Canonical SMILES	ESOL Log S	ESOL Solubility (mg/ml)	ESOL Solubility (mol/l)	ESOL Class	Ali Log S	Ali Solubility (mg/ml)	Ali Solubility (mol/l)	Ali Class	Silicos-IT LogSw	Silicos-IT Solubility (mg/ml)	Silicos-IT Solubility (mol/l)	Silicos-IT class
**var1**	O=C(c1cccc2n1nc(c2)C(C)C)NC[C@@H]1CCN(CC1)[C@@H]1CCC[C@H]2[C@H]1CCOC2	-4.91	5.39E-03	1.23E-05	Moderately soluble	-4.97	4.71E-03	1.07E-05	Moderately soluble	-5.24	2.52E-03	5.75E-06	Moderately soluble
**var2**	COC[C@H]1CCC[C@H](C1)N1CC[C@H](CC1)CNC(=O)c1cccc2n1nc(c2)C(C)C	-4.69	8.68E-03	2.03E-05	Moderately soluble	-4.94	4.92E-03	1.15E-05	Moderately soluble	-5.44	1.53E-03	3.60E-06	Moderately soluble
**var3**	OC[C@H]1CCC[C@H](C1)N1CC[C@H](CC1)CNC(=O)c1cccc2n1nc(c2)C(C)C	-4.34	1.90E-02	4.59E-05	Moderately soluble	-4.61	1.02E-02	2.46E-05	Moderately soluble	-4.75	7.27E-03	1.76E-05	Moderately soluble
**var4**	OC/C=C/CN1CC[C@H](CC1)CNC(=O)c1cccc2n1nc(c2)C(C)C	-3.27	2.00E-01	5.41E-04	Soluble	-3.34	1.68E-01	4.54E-04	Soluble	-4.08	3.10E-02	8.36E-05	Moderately soluble
**var5**	O=CCC[C@@H]1OCCN(C1)CCCCNC(=O)c1cccc2n1nc(c2)C(C)C	-3.13	2.96E-01	7.40E-04	Soluble	-3.29	2.04E-01	5.08E-04	Soluble	-5.16	2.75E-03	6.87E-06	Moderately soluble
**var6**	O=C(c1cccc2n1nc(c2)C(C)C)NC[C@@H]1CCN(CC1)C[C@H]1CCOCO1	-3.74	7.26E-02	1.81E-04	Soluble	-3.7	7.99E-02	1.99E-04	Soluble	-4.49	1.29E-02	3.21E-05	Moderately soluble
**var7**	O=C(c1cccc2n1nc(c2)C(C)C)NCCCCN1CCO[C@H](C1)C1CCOCC1	-3.93	5.01E-02	1.17E-04	Soluble	-3.97	4.59E-02	1.07E-04	Soluble	-5.28	2.26E-03	5.27E-06	Moderately soluble
**var8**	CC(=O)CNCCCCNC(=O)c1cccc2n1nc(c2)C(C)C	-2.68	6.84E-01	2.07E-03	Soluble	-3.08	2.77E-01	8.37E-04	Soluble	-5.29	1.68E-03	5.08E-06	Moderately soluble
**var9**	COC[C@H]1CCC[C@H](C1)N1CC[C@@H](C[C@@H]1C(=O)C)CNC(=O)c1cccc2n1nc(c2)C(C)C	-4.73	8.75E-03	1.87E-05	Moderately soluble	-5.07	4.00E-03	8.54E-06	Moderately soluble	-5.51	1.46E-03	3.11E-06	Moderately soluble
**var10**	CC(=O)[C@@H]1NCC[C@@H](C1)CNC(=O)c1cccc2n1nc(c2)C(C)C	-3.06	3.01E-01	8.80E-04	Soluble	-3.15	2.43E-01	7.08E-04	Soluble	-4.59	8.72E-03	2.55E-05	Moderately soluble

S4. References

1. Brodney, M.A.; Johnson, D.E.; Sawant-Basak, A.; Coffman, K.J.; Drummond, E.M.; Hudson, E.L.; Fisher, K.E.; Noguchi, H.; Waizumi, N.; McDowell, L.L.; et al. Identification of Multiple 5-HT $_4$ Partial Agonist Clinical Candidates for the Treatment of Alzheimer's Disease. *J. Med. Chem.* **2012**, *55*, 9240–9254, doi:10.1021/jm300953p.
2. Nirogi, R.; Mohammed, A.R.; Shinde, A.K.; Bogaraju, N.; Gagginapalli, S.R.; Ravella, S.R.; Kota, L.; Bhyrapuneni, G.; Muddana, N.R.; Benade, V.; et al. Synthesis and SAR of Imidazo[1,5-a]pyridine derivatives as 5-HT$_4$ receptor partial agonists for the treatment of cognitive disorders associated with Alzheimer's disease. *Eur. J. Med. Chem.* **2015**, *103*, 289–301, doi:10.1016/j.ejmech.2015.08.051.
3. Nirogi, R.; Mohammed, A.R.; Shinde, A.K.; Gagginapally, S.R.; Kancharla, D.M.; Middekadi, V.R.; Bogaraju, N.; Ravella, S.R.; Singh, P.; Birangal, S.R.; et al. Synthesis, Structure–Activity Relationships, and Preclinical Evaluation of Heteroaromatic Amides and 1,3,4-Oxadiazole Derivatives as 5-HT 4 Receptor Partial Agonists. *J. Med. Chem.* **2018**, *61*, 4993–5008, doi:10.1021/acs.jmedchem.8b00457.
4. Hawkins, P.C.D.; Skillman, A.G.; Warren, G.L.; Ellingson, B.A.; Stahl, M.T. Conformer Generation with OMEGA: Algorithm and Validation Using High Quality Structures from the Protein Databank and Cambridge Structural Database. *J. Chem. Inf. Model.* **2010**, *50*, 572–584, doi:10.1021/ci100031x.
5. Paul C. D. Hawkins, *; A. Geoffrey Skillman, and; Nicholls, A. Comparison of Shape-Matching and Docking as Virtual Screening Tools. **2006**, doi:10.1021/JM0603365.
6. Golbraikh, A.; Tropsha, A. Beware of q2! *J. Mol. Graph. Model.* **2002**, *20*, 269–276, doi:10.1016/S1093-3263(01)00123-1.
7. Rácz, A.; Bajusz, D.; Héberger, K. Consistency of QSAR models: Correct split of training and test sets, ranking of models and performance parameters. *SAR QSAR Environ. Res.* **2015**, *26*, 683–700, doi:10.1080/1062936X.2015.1084647.
8. Ferreira, L.L.G.; Andricopulo, A.D. ADMET modeling approaches in drug discovery. *Drug Discov. Today* **2019**, *24*, 1157–1165, doi:10.1016/j.drudis.2019.03.015.
9. Pires, D.E.V.; Blundell, T.L.; Ascher, D.B. pkCSM: Predicting small-molecule pharmacokinetic and toxicity properties using graph-based signatures. *J. Med. Chem.* **2015**, *58*, 4066–4072, doi:10.1021/acs.jmedchem.5b00104.
10. Daina, A.; Michielin, O.; Zoete, V. SwissADME: A free web tool to evaluate pharmacokinetics, drug-likeness and medicinal chemistry friendliness of small molecules. *Sci. Rep.* **2017**, *7*, 1–13, doi:10.1038/srep42717.
